# Supplementary material for: The motion sensitivity and predictive utility of different estimates of interregional functional coupling in resting-state functional MRI
Source: Netw Neurosci. 2026 Mar 20;10(1):221–43. doi: 10.1162/NETN.a.534 (PMC13008380; doi:10.1162/NETN.a.534)
Supplement: Supplementary file 1 [file netn-10-1-221-s001.pdf]

The Motion Sensitivity and Predictive Utility of Different Estimates of Inter-regional Functional Coupling in Resting-state Functional MRI. *– Supplementary Material*

1. Matched Sparsity analysis.

Given that both rDCM and GLASSO partial correlations inherently impose sparsity constraints on connectivity matrices, we conducted a supplementary analysis where all other metrics were thresholded to match the sparsity level of the GLASSO-derived matrices. Specifically, for each participant, we retained only the top X% of connections (where X corresponded to the connection density preserved in the GLASSO output). Such that, if GLASSO retained the top 10% of connections in the matrix, we kept the top 10% of the highest connections in the correlation, mutual information (MI), and coherence measures. As PID retains synergistic and redundant components only, we did not control for sparsity in these matrices.

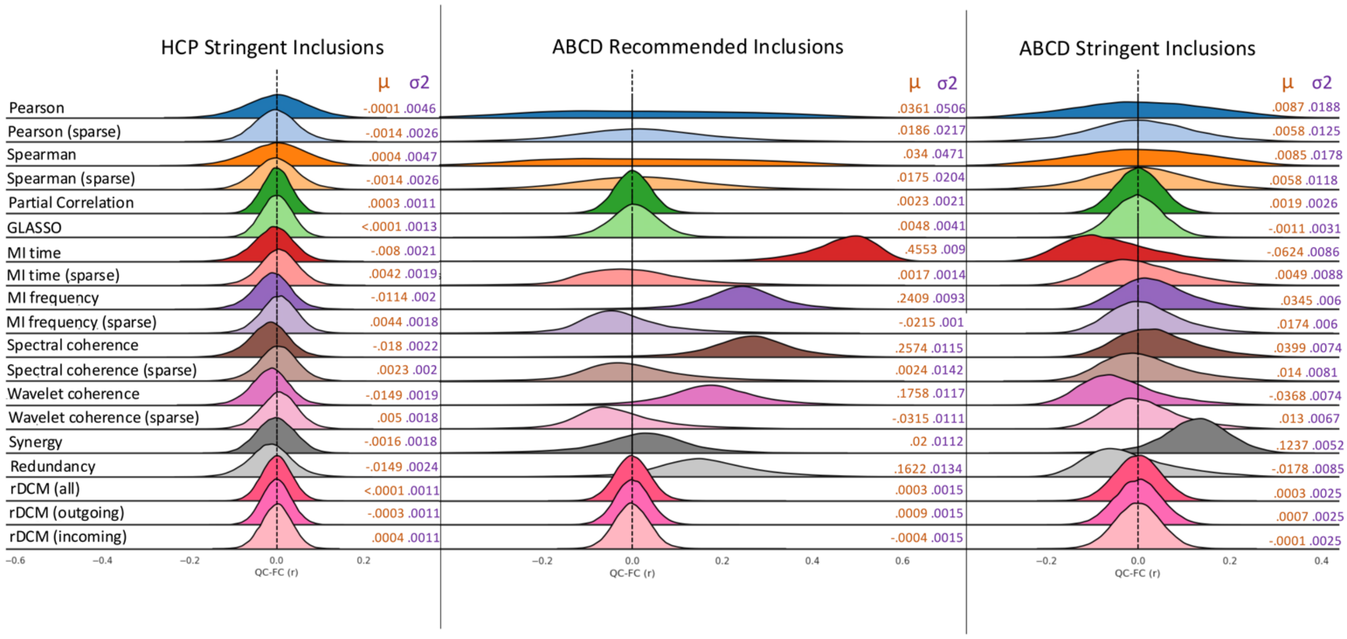

Supplementary Figure 1. Distribution of QC-FC correlations across all functional connections presented in the main text and after sparsity constraints were imposed. Distribution plots compare motion profiles for HCP participants meeting stringent motion criteria, ABCD participants passing standard quality control inclusion criteria, and ABCD participants meeting stringent motion exclusion thresholds. Measures are ordered as: Correlation → Partial correlation → Mutual Information → Coherence → PID → rDCM.

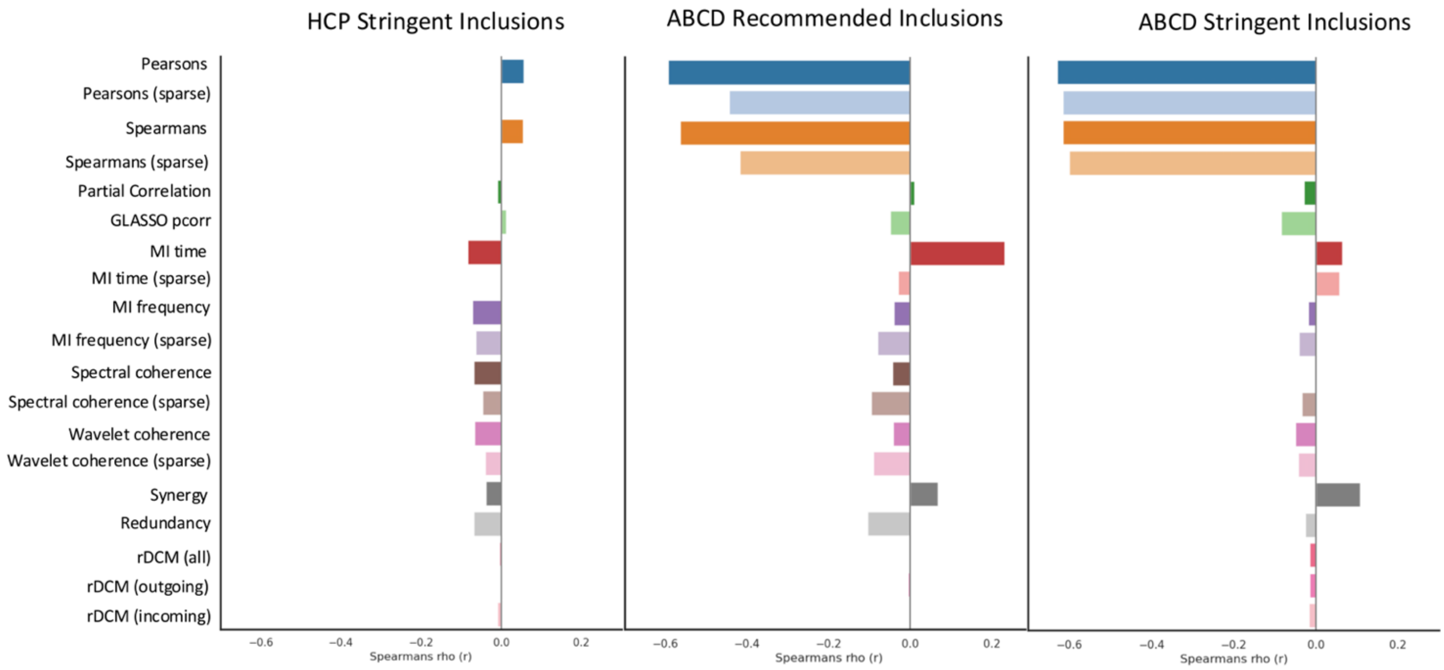

Supplementary Figure 2. QC-FC Distance Dependent correlations presented in the main text, including results after sparsity constraints were imposed. Bars show the distance dependent value for each connectivity metric for HCP participants meeting stringent motion criteria, ABCD participants passing standard quality control inclusion criteria, and ABCD participants meeting stringent motion exclusion thresholds. Measures are ordered as: Correlation → Partial correlation → Mutual Information → Coherence → PID → rDCM.

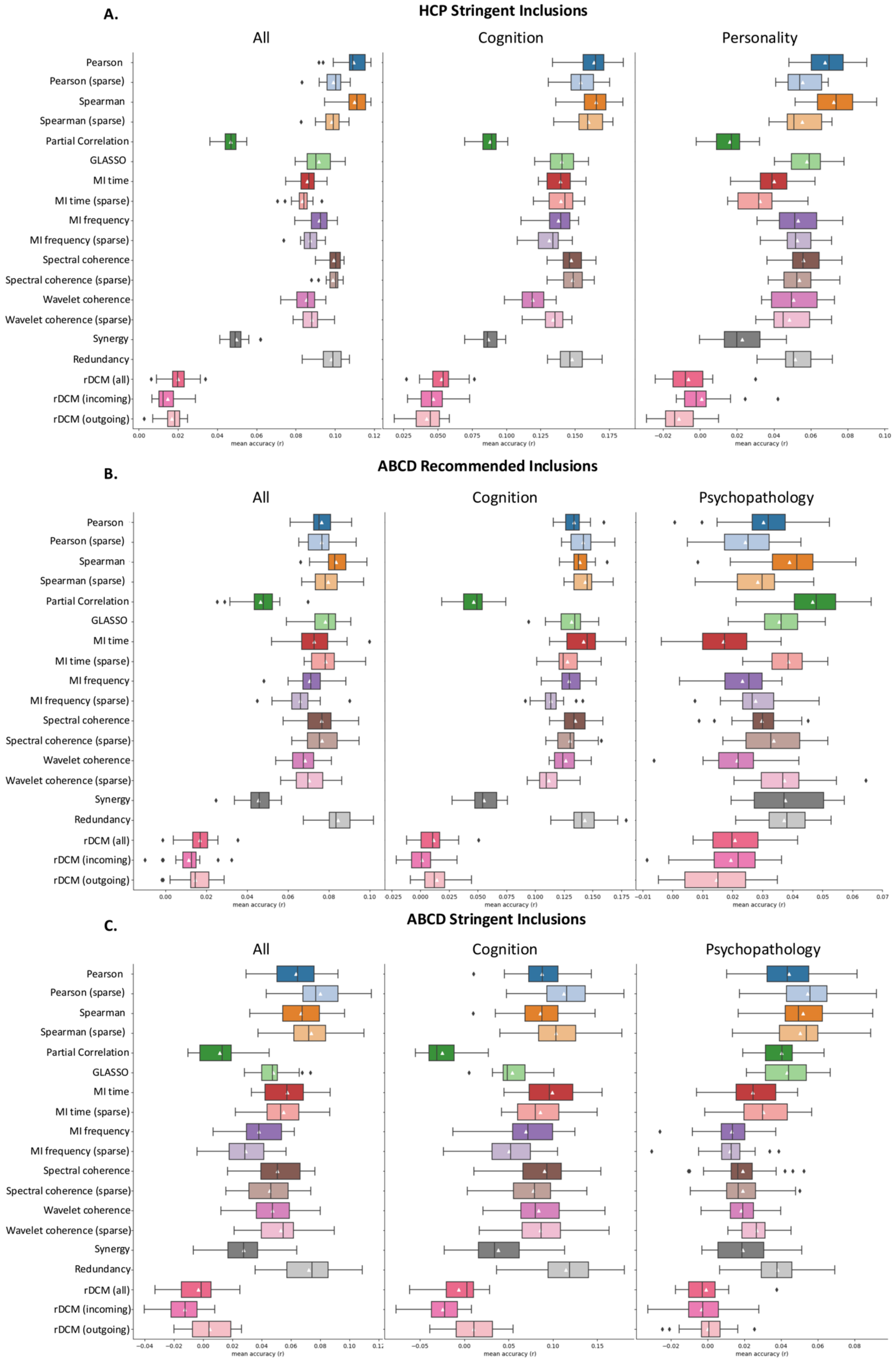

Supplementary Figure 3. KRR prediction accuracies presented in the main text, including results after

sparsity constraints were imposed. The boxplots show the median and interquartile ranges for accuracies averaged over cross validation folds, repetitions, and behaviours. Mean predictive accuracy for each connectivity measure is shown with a white triangle. Prediction accuracies are shown for HCP participants meeting stringent motion criteria, ABCD participants passing standard quality control inclusion criteria, and ABCD participants meeting stringent motion exclusion thresholds. Measures are ordered as: Correlation → Partial correlation → Mutual Information → Coherence → PID → rDCM.

2. *Behavioural Variable Details*

Supplementary Table 1. Behavioural variables used in the KRR for the HCP dataset. For each variable, the corresponding field names in the HCP behavioural csv file are shown together with whether they belonged to the cognition, personality, or other domains. The table has been adapted from Li et al. (2019).

Description	*Name in the HCP dataset*	*Cognitive*	*Personality*	*Others*
Visual Episodic Memory	PicSeq_Unadj_Score	✓		
Cognitive Flexibility (DCCS)	CardSort_Unadj	✓		
Inhibition (Flanker Task)	Flanker_Unadj	✓		
Fluid Intelligence (PMAT)	PMAT24_A_CR	✓		
Vocabulary (Pronunciation)	ReadEng_Unadj	✓		
Vocabulary (Picture Matching)	PicVocab_Unadj	✓		
Processing Speed	ProcSpeed_Unadj	✓		
Delay Discounting	DDisc_AUC_40K	✓		
Spatial Orientation	VSPLOT_TC	✓		
Sustained Attention – Sens.	SCPT_SEN	✓		
Sustained Attention – Spec.	SCPT_SPEC	✓		
Verbal Episodic Memory	IWRD_TOT	✓		
Working Memory (List Sorting)	ListSort_Unadj	✓		
Cognitive Status (MMSE)	MMSE	✓		
Sleep Quality (PSQI)	PSQI_Score			✓
Walking Endurance	Endurance_Unadj			✓
Walking Speed	GaitSpeed_Comp			✓
Manual Dexterity	Dexterity_Unadj			✓
Grip Strength	Strength_Unadj			✓

Odour Identification	Odor_Unadj			✓
Pain Interference Survey	PainInterf_Tscore			✓
Taste Intensity	Taste_Unadj			✓
Contrast Sensitivity	Mars_Final			✓
Emotional Face Matching	Emotion_Task_Face_Acc			✓
Arithmetic	Language_Task_Math_Avg_Difficulty_Level	✓		
Story Comprehension	Language_Task_Story_Avg_Difficulty_Level	✓		
Relational Processing	Relational_Task_Acc			✓
Social Cognition – Random	Social_Task_Perc_Random			✓
Social Cognition – Interaction	Social_Task_Perc_TOM			✓
Working Memory (N-back)	WM_Task_Acc	✓		
Agreeableness (NEO)	NEOFAC_A		✓	
Openness (NEO)	NEOFAC_O		✓	
Conscientiousness (NEO)	NEOFAC_C		✓	
Neuroticism (NEO)	NEOFAC_N		✓	
Extraversion (NEO)	NEOFAC_E		✓	
Emotion Recognition – Total	ER40_CR			✓
Emotion Recognition – Angry	ER40ANG			✓
Emotion Recognition – Fear	ER40FEAR			✓
Emotion Recognition – Happy	ER40HAP			✓
Emotion Recognition - Neutral	ER40NOE			✓
Emotion Recognition – Sad	ER40SAD			✓
Anger – Affect	AngAffect_Unadj		✓	

Anger – Hostility	AngHostil_Unadj		✓	
Anger – Aggression	AngAggr_Unadj		✓	
Fear – Affect	FearAffect_Unadj		✓	
Fear – Somatic Arousal	FearSomat_Unadj		✓	
Sadness	Sadness_Unadj		✓	
Life Satisfaction	LifeSatisf_Unadj			✓
Meaning & Purpose	MeanPurp_Unadj			✓
Positive Affect	PosAffect_Unadj		✓	
Friendship	Friendship_Unadj			✓
Loneliness	Loneliness_Unadj			✓
Perceived Hostility	PercHostil_Unadj			✓
Perceived Rejection	PercReject_Unadj			✓
Emotional Support	EmotSupp_Unadj			✓
Instrument Support	InstruSupp_Unadj			✓
Perceived Stress	PercStress_Unadj			✓
Self-Efficacy	SelfEff_Unadj			✓

Supplementary Table 2. Behavioural variables used in the KRR for the ABCD dataset. For each variable, the corresponding field names in the ABCD behavioural csv file are shown together with whether they belonged to the cognition or psychopathology domains. The table has been adapted from Li et al. (2019).

Description	*Name in the ABCD dataset*	*Cognitive*	*Psychopathology*
Vocabulary	nihtbx_picvocab_uncorrected	✓	
Attention	nihtbx_flanker_uncorrected	✓	
Working Memory	nihtbx_list_uncorrected	✓	
Executive Function	nihtbx_cardsort_uncorrected	✓	
Processing Speed	nihtbx_pattern_uncorrected	✓	
Episodic Memory	nihtbx_picture_uncorrected	✓	
Reading	nihtbx_reading_uncorrected	✓	
Fluid Cognition	nihtbx_fluidcomp_uncorrected	✓	
Crystalised Cognition	nihtbx_cryst_uncorrected	✓	
Overall Cognition	nihtbx_totalcomp_uncorrected	✓	
Short Delay Recall	pea_ravlt_sd_trial_vi_tc	✓	

Long Delay Recall	pea_ravlt_ld_trial_vii_tc	✓	
Fluid Intelligence	Pea_wiscv_trs	✓	
Visuospatial Accuracy	lmt_scr_perc_correct	✓	
Visuospatial reaction time	lmt_scr_rt_correct	✓	
Visuospatial Efficiency	lmt_scr_efficiency	✓	
Negative Urgency	upps_y_ss_negative_urgency		✓
Lack of Planning	upps_y_ss_lack_of_planning		✓
Sensation Seeking	upps_y_ss_sensation_seeking		✓
Positive Urgency	upps_y_ss_positive_urgency		✓
Lacks Perseverance	upps_y_ss_lack_of_perserverance		✓
Behavioural Inhibition	bis_y_ss_bis_sum		✓
Reward Responsiveness	bis_y_ss_bar_rr		✓
Drive	bis_y_ss_bas_drive		✓
Fun Seeking	bis_y_ss_bas_fs		✓
Anxious Depressed	cbcl_scr_syn_anxdep_r		✓
Withdrawn Depressed	cbcl_scr_syn_withdep_r		✓
Somatic Complaints	cbcl_scr_syn_somatic_r		✓
Social Problems	cbcl_scr_syn_social_r		✓
Thought Problems	cbcl_scr_syn_thought_r		✓
Attention Problems	cbcl_scr_syn_attention_r		✓
Rule Breaking	cbcl_scr_syn_rulebreak_r		✓
Aggression	cbcl_scr_syn_aggresssive_r		✓
Total Psychosis Symptoms	pps_y_ss_number		✓
Psychosis Severity	pps_y_ss_severity_score		✓
Mania	pgbi_p_ss_score		✓
